# DEK is a potential marker for aggressive phenotype and irinotecan-based therapy response in metastatic colorectal cancer

**DOI:** 10.1186/1471-2407-14-965

**Published:** 2014-12-16

**Authors:** Javier Martinez-Useros, Maria Rodriguez-Remirez, Aurea Borrero-Palacios, Irene Moreno, Arancha Cebrian, Teresa Gomez del Pulgar, Laura del Puerto-Nevado, Ricardo Vega-Bravo, Alberto Puime-Otin, Nuria Perez, Sandra Zazo, Clara Senin, Maria J Fernandez-Aceñero, Maria S Soengas, Federico Rojo, Jesus Garcia-Foncillas

**Affiliations:** Translational Oncology Division, OncoHealth Institute, Health Research Institute - University Hospital “Fundación Jiménez Díaz”-UAM, Av. Reyes Católicos 2, 28040 Madrid, Spain; Department of Pathology, University Hospital “Fundación Jiménez Díaz”-UAM, Madrid, Spain; Department of Oncology, Vigo Hospital, Vigo, Spain; Melanoma Research Group, Spanish National Cancer Research Centre, Madrid, Spain; Department of Pathology, Clinico San Carlos University Hospital, Madrid, Spain

**Keywords:** DEK, Irinotecan, Aggressive phenotype, Metastatic colorectal cancer, KRAS

## Abstract

**Background:**

DEK is a transcription factor involved in stabilization of heterochromatin and cruciform structures. It plays an important role in development and progression of different types of cancer. This study aims to analyze the role of DEK in metastatic colorectal cancer.

**Methods:**

Baseline DEK expression was firstly quantified in 9 colorectal cell lines and normal mucosa by WB. SiRNA-mediated DEK inhibition was carried out for transient DEK silencing in DLD1 and SW620 to dissect its role in colorectal cancer aggressiveness. Irinotecan response assays were performed with SN38 over 24 hours and apoptosis was quantified by flow cytometry. *Ex-vivo* assay was carried out with 3 fresh tumour tissues taken from surgical resection and treated with SN38 for 24 hours. DEK expression was determined by immunohistochemistry in 67 formalin-fixed paraffin-embedded tumour samples from metastatic colorectal cancer patients treated with irinotecan-based therapy as first-line treatment.

**Results:**

The DEK oncogene is overexpressed in all colorectal cancer cell lines. Knock-down of DEK on DLD1 and SW620 cell lines decreased cell migration and increased irinotecan-induced apoptosis. In addition, low DEK expression level predicted irinotecan-based chemotherapy response in metastatic colorectal cancer patients with *KRAS* wild-type.

**Conclusions:**

These data suggest DEK overexpression as a crucial event for the emergence of an aggressive phenotype in colorectal cancer and its potential role as biomarker for irinotecan response in those patients with *KRAS* wild-type status.

## Background

Colorectal cancer (CRC) is one of the most common gastrointestinal malignant tumors in the world and it has one of the highest rates of morbidity and mortality worldwide. There are about 1.36 million new-onset patients around the world each year, and 0.7 million CRC patients died of it in 2012 [1]. The 5-year survival rate for colorectal cancer is approximately 55% because of its invasion and metastasis.

The first-line treatment of metastatic colorectal cancer (mCRC) is based on fluoropyrimidines (5-fluorouracil/folinic acid) given in combination with the prodrugs oxaliplatin [2–4] and/or irinotecan [5–9].

The active metabolite of irinotecan, SN38, inhibits topoisomerase I and prevents DNA from unwinding [10]. Topoisomerase I expression has correlated with irinotecan response in several studies [11, 12] but this procedure is not currently performed as part of the selection of therapy for mCRC.

DEK was identified as a fusion protein with the CAN nucleoporin due to the translocation t(6;9) in a subtype of acute myeloid leukaemia [13]. It was later described as a transcription factor overexpressed in multiple neoplasms including bladder cancer [14], breast cancer [15], glioblastoma [16], hepatocellular carcinoma [17], melanoma [18], retinoblastoma [19, 20], colorectal cancer [21, 22] and other types of cancer, such as oral, ovarian, or uterine cervical cancer [21, 23–25].

It has been reported that *DEK* promoter is regulated by E2F1 [21], and its activation leads to transcription of *DEK* mRNA. Functionally, DEK is involved in the DNA repair machinery through interaction with PARP-1 [26], suppresses cellular senescence, apoptosis, differentiation, and promotes transformation *in vitro* and *in vivo* [27–29]. Furthermore, DEK has been suggested as a potential marker for bladder cancer [14], an independent predictor for prognosis in colorectal cancer patients (stages I-III) [22] and a specific marker to neoadjuvant chemotherapy for breast cancer [30].

In this study, we analyze the oncogenic role of DEK in CRC cell lines. As well as, we propose its potential use as a marker of irinotecan-based chemotherapy response in metastatic colorectal cancer patients.

This new function of DEK settles this oncogene as a potential marker for clinical practice, as only 20% to 30% of patients with mCRC respond to irinotecan-based therapy in first-line treatment. The applicability of DEK as a tool for improved decision-making in routine diagnostic assessment requires further validation.

## Methods

### Cell lines

Nine human-derived CRC cell lines obtained from the American Type Culture Collection (SW620 (CCL-227) and LOVO (CCL-229) from metastatic foci origin; DLD1 (CCL-221), SW480 (CCL-228), RKO (CRL-2577), WIDR (CCL-218), LS513 (CRL-2134), HCT15 (CCL-225), and HCT116 (CCL-247) from primary tumor origin) were cultured with RPMI (Gibco) supplemented with 10% FBS (Gibco), penicillin (100 U/mL)/streptomycin (100 U/mL) (Invitrogen, Life Technologies). Two human colon mucosa from frozen tissue were used as controls.

### Patient samples

A total of 67 mCRC patients who received FOLFIRI regimen as first-line treatment were collected for the study. *KRAS* mutation status was determined with Cobas® *KRAS* Mutation Test (Roche Diagnostics) that offers broad mutation coverage of *KRAS* codons 12, 13 and 61. We found 35 patients with *KRAS*^wt^, 26 patients with *KRAS*^mut^ status and 6 could not be determined because the quality of DNA was not enough. The clinical-pathological features of the 67 patients included in the study are summarized in Table 1.

**Table 1:** **Clinical features of metastatic colorectal cancer patients treated with irinotecan-based therapy**

Characteristics	Patients (N = 67)
**Median age-years (range)**	62 (33–79)
**Sex**	
Male	47 (70%)
Female	20 (30%)
**Median CEA (range, ng/mL)**	16 (0–2066)
**Performance status WHO**	
0	29 (43%)
1	35 (52%)
2	3 (5%)
**Site of primary tumor**	
Colon	35 (52%)
Rectum	32 (48%)
**METASTASIS**	
Liver	31 (47%)
Liver & other	19 (28%)
Other	15 (22%)
N.A.	2 (3%)
**KRAS**	
Wild-type	35 (52%)
Mutated	26 (39%)
N.A.	6 (9%)
**BRAF**	
Wild-type	60 (90%)
Mutated	7 (10%)
**Biologic treatment**	
Bevacizumab	23 (34%)
Cetuximab	7 (11%)
None	37 (55%)
**TOPO I expression level**	
High	21 (31%)
Low	20 (30%)
N.A.	26 (39%)
**DEK expression level**	
High	21 (31%)
Low	46 (69%)

N.A.: not available. Other refers to lung, lymph node and/or peritoneal metastasis.

Clinical samples used in the study were kindly supplied from the BioBank of the Fundacion Jimenez Diaz-Universidad Autonoma de Madrid (RD09/0076/00101 - Spain). This study has been evaluated by The Ethics Committee of Clinical Research of Fundacion Jimenez Diaz (act number 17/14).

### *Ex-vivo*assay

*Ex-vivo* assays were designed to predict the sensitivity or resistance of a set of tumors to irinotecan. To perform these assays, three tumor samples from 3 different patients were taken after surgical resection. Each sample was divided in two pieces and transferred onto a 12-well plate and cultured in DMEM (Gibco) supplemented with 10% FBS, penicillin (100 U/mL)/streptomycin (100 U/mL). One of the tumor pieces was treated with SN38 (5 nM) (Sigma-Aldrich), whereas the other half remained untreated. After 24 hours, the tissues were processed for IHC.

### Western blot

Total protein from CRC cell lines and normal mucosa was extracted with RIPA buffer supplemented with protease inhibitor cocktail (Roche). Samples were fractionated by SDS–polyacrylamide gel electrophoresis, transferred to nitrocellulose membranes (Biorad), and proteins were detected using specific antibodies for DEK (610948, BD Biosciences), cleaved-Caspase-3 (9664, Cell Signaling) and actin (a1978, Sigma-Aldrich). Horseradish peroxidase-linked sheep anti-mouse (NA931V) antibodies (GE-Healthcare) were used as the secondary antibodies. Blots were developed with the Amersham ECL Prime Western Blotting Detection Reagent (GE-Healthcare).

### DEK silencing

Three different siRNAs for DEK were used (Silencer Select Pre-designed siRNA s15457, s15458, and s15459) (Ambion, Life Technologies). Gene silencing was performed with 3.5 million cells from two different CRC cell lines, DLD1 and SW620, by transfecting 600 pmol of each siRNA or the Silencer Negative Control-1 siRNA (Ambion, Life Technologies) using Lipofectamine 2000 reagent (Invitrogen, Life Technologies).

### Cell viability, apoptosis, and cell cycle

Cell viability was determined using the 3-(4,5-dimethyl-thiazol-2yl)-5-(3-carboxymethoxyphenyl)-2-(4-sulfophenyl)-2H-tetrazolium (MTS) reduction assay (Promega).

Apoptosis and cell cycle were analyzed after DEK silencing and treatment for 24 hours with the known IC_50_ dose of active principle of irinotecan (SN38, 50 nM) [31], oxaliplatin (LOHP, 1 μM) [32] and 5-fluorouracil (5FU, 1 μM) [33]. Apoptosis was assessed using the Annexin-V-FITC Apoptosis Detection Kit (BD Biosciences) according to the manufacturer’s protocol. For cell cycle analysis, cells were collected by centrifugation, fixed with pre-cooled 70% ethanol for 2 h, incubated with 0.5 mg/mL RNase (Sigma-Aldrich) at 37°C for 30 min, and stained with propidium bromide (BD Biosciences). Fluorescence was detected on a FACSCanto II flow cytometer (BD Biosciences) and analyzed with FACSDiva software (BD Biosciences). All experiments were performed in triplicate.

### Wound healing and Boyden chamber migration assay

Cell motility after DEK downregulation was estimated by wound healing assays. Cells were grown as a monolayer and an artificial homogenous wound was created with a sterile plastic 10 μL micropipette tip. The growth of cells in the wound was measured at 6, 12, and 24 hours.

Migration assays were performed in cell culture inserts with 8-μm pores in 24-well plates (Transwells, BD Biosciences). DLD1 and SW620 cells were seeded at a density of 5×10^4^ cells per insert in 300 μl RPMI. The recipient wells received 750 μl RPMI supplemented with 20% FBS. The migration was determinated after 24 h. Afterwards, cells were fixed and stained with toluidine blue (Sigma-Aldrich). The non-migrated cells on the upper side of the membrane were removed with a cotton swab. On each membrane, the cells of 10 randomly selected fields (10X objective) were counted, and the mean number of cells per visual field was determined. The migration index was determined as migrated cells ratio relative to siRNA control transfected cells. Three independent experiments were done and all experiments were performed in triplicate wells.

### Immunohistochemistry

Immunohistochemical staining was conducted in formalin-fixed paraffin-embedded (FFPE) tumor sections. Biopsies were cut and incubated with PT-Link (Dako) for 20 min at 95°C in a high pH buffered solution (EnVision Dako kit). To block endogenous peroxidase holders were incubated with peroxide (EnVision Flex peroxidase-blocking reagent). Biopsies were stained for 20 min with a 1:50 dilution of DEK antibody (610948, BD Biosciences), 1:100 of cleaved-Caspase-3 (9664, Cell Signaling), 1:150 of Ki-67 (clone SP6, Master Diagnostica) or 1:500 of Topoisomerase I (NBP1-95632, Novus Biologicals) followed by incubation with the appropriate anti-Ig horseradish peroxidase-conjugated polymer (EnVision, Dako) to detect antigen-antibody. Sections were then visualized with 3,3’-diaminobenzidine as a chromogen for 5 min and counterstained with haematoxylin.

Immunoreactivity was scored semiquantitatively for both the intensity and the proportion of cell staining. A HistoScore (HScore) was calculated as the percentage of cells positively stained with low, medium or high staining intensity. The final score was determined after applying a weighting factor to each estimate. The following formula was used: HScore = (low%) × 1 + (medium%) × 2 + (high%) × 3 and the results ranged from 0 to 300.

### Statistical analysis

Mann–Whitney test was used to compare differences between groups.

Demographic and baseline characteristics of mCRC patients included in the study were summarized by descriptive statistics.

Statistical association between DEK expression and progression-free survival was assessed. Patients were divided into expression groups (tertiles: low, medium, high) based on DEK levels. The third tertile was established as the cut-off point, leaving low- and high-risk patient groups. In the case of topoisomerase I, patients were stratified in low- or high-risk groups using the median as cut-off point. Survival curves were estimated using the Kaplan-Meier method and significant survival differences between groups were determined by the log-rank test.

Univariate and multivariate Cox proportional-hazards analyses were used to assess the association between DEK expression and patient survival. In the multivariate analysis only those variables that were statistically significant in the univariate analysis were considered. A *P* value <0.05 indicated statistical significance. All statistics were performed with the IBM SPSS statistics 20.0.

## Results

### DEK downregulation significantly decreased cell viability and migration

**Figure 1:**
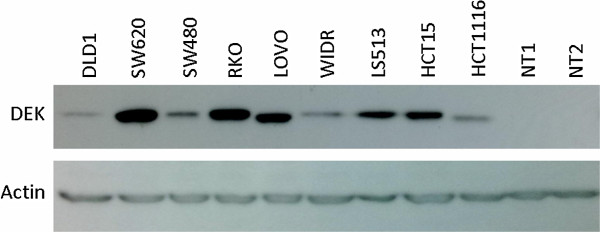
**DEK is overexpressed in CRC.** Western blot analysis of a panel of human derived colorectal cancer cell lines showed higher DEK expression than human non-tumor mucosa tissues (NT1, NT2).

DEK protein levels were analyzed in a panel of 9 human-derived CRC cell lines and compared with the expression in 2 non-tumor mucosa tissues. All tested cell lines showed high DEK expression levels compared to mucosa tissues (Figure 1). It is highlighting that two out of the three cell lines showing the highest DEK expression levels are those with metastatic origin (SW620 and LOVO).

**Figure 2:**
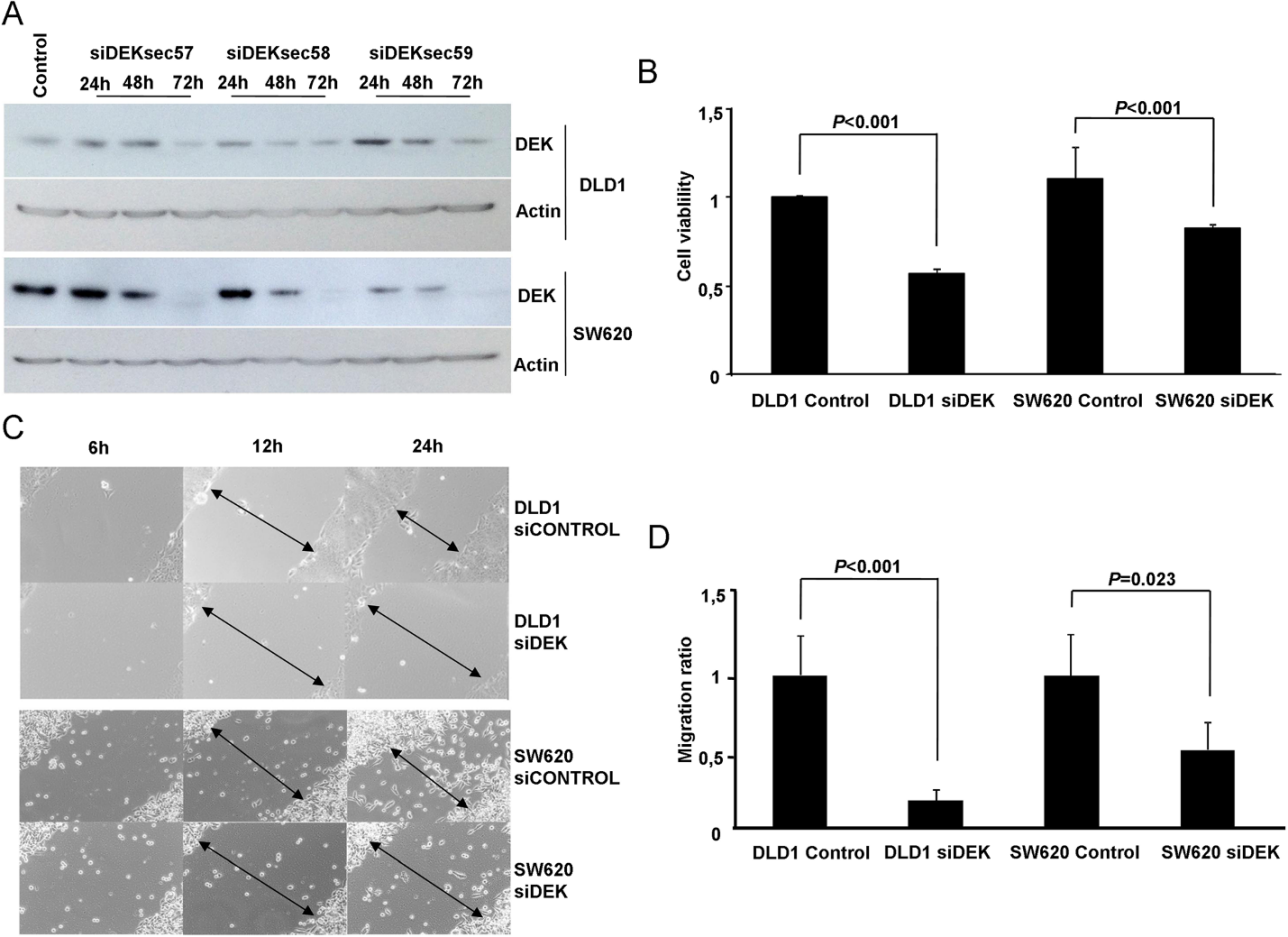
**DEK downregulation decreases cell viability, migration and invasion. A)** Three different siRNAs of DEK (siDEKsec57, sec58 and sec59) were used to downregulate DEK protein expression in DLD1 and SW620 cell lines. We verified DEK expression levels by western blot at 24, 48, and 72 hours after transfection. **B)** MTS assay showed that both DLD1 and SW620 cell lines decreased cell viability at 72 hours after DEK downregulation (*P* < 0.001). **C)** Microscope images of wound healing assay showed a reduced cell migration after DEK downregulation in both cell lines. Images are representative from one experiment and were taken at 6, 12, and 24 hours after scratching. Arrows represent distance between cell migration heads. **D)** Cell invasion assays performed in Boyden chamber showed that DEK silencing decreased invasion ability of both cell lines. All assays were performed with 2 different siRNA sequences (siDEKsec57 and 58).

To assess whether DEK is involved in aggressive phenotype, we selected DLD1 cell line derived from the primary tumor and SW620 derived from a metastatic focus, due to the different origin and different DEK expression pattern.To downregulate DEK expression, 3 different siRNA sequences were used to transfect DLD1 and SW620 cells. Proteins were extracted at 24, 48, and 72 hours after transfection. DEK downregulation was confirmed at protein level by showing a decreased expression from 48 to 72 hours in both cell lines (Figure 2A).

We observed that when DEK was silenced, cell viability significantly decreased in both cell lines (*P* < 0.001) (Figure 2B).

We then aimed to analyze whether downregulation of DEK affects migration of DLD1 and SW620 cell lines. When wound healing assays were performed, a delay of 12 to 24 hours was observed in DEK silenced cells compared to control (Figure 2C). In addition, we observed a significant reduction in migration ability of both cell lines, being higher on DLD1 (*P* < 0.001) than SW620 (*P* = 0.023) (Figure 2D).

### Low DEK expression sensitized to SN38

Silenced DEK cell lines were cultured in the presence of oxaliplatin active principle, LOHP, irinotecan active principle, SN38, and 5FU. 72 hours after DEK downregulation, and 24 hours after treatments, cell cycle and apoptosis were assessed. No significant effect was observed in the cell cycle analysis (data not shown).

**Figure 3:**
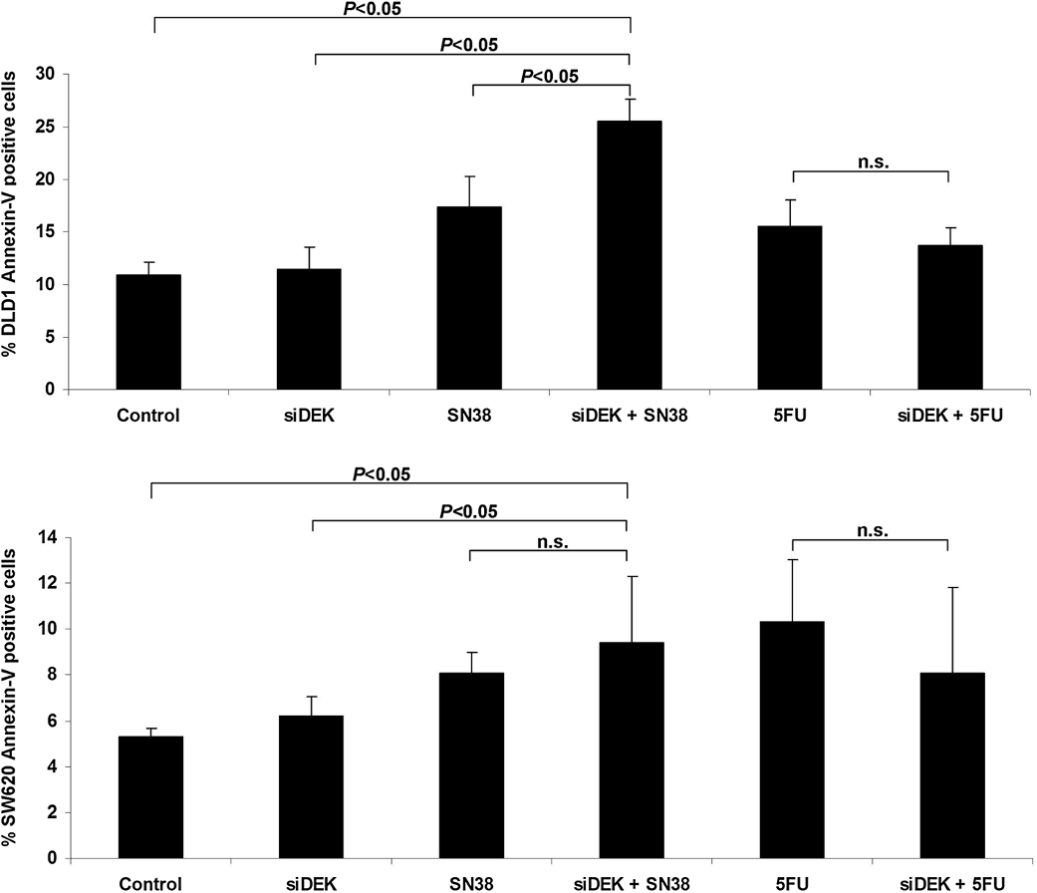
**Irinotecan response is increased when DEK is silenced.** Diagram shows percentage of apoptotic cells stained with Annexin-V after treatments. DLD1 and SW620 cell lines were DEK knocked-down and treated with SN38 or 5FU separately and compared with control cells (control siRNA and untreated). Combination of siDEK and SN38 increased apoptosis (*P* < 0.05) compared to control or DEK silenced in both cell lines. In addition, combination increased apoptosis (*P* < 0.05) compared to single SN38 treatment in DLD1 cell line. No significant differences were observed after 5FU treatment. Results are expressed as the average of downregulation with 2 different siRNA sequences (siDEKsec57 and 58) in triplicate.

DEK downregulation was not enough to produce significant annexin-V induction. However when it was combined with SN38, annexin-V levels significantly increased in both cell lines (*P* < 0.05) (Figure 3). This effect was not observed when DEK silencing was combined with 5FU treatment (Figure 3) or LOHP treatment (data not shown).

**Figure 4:**
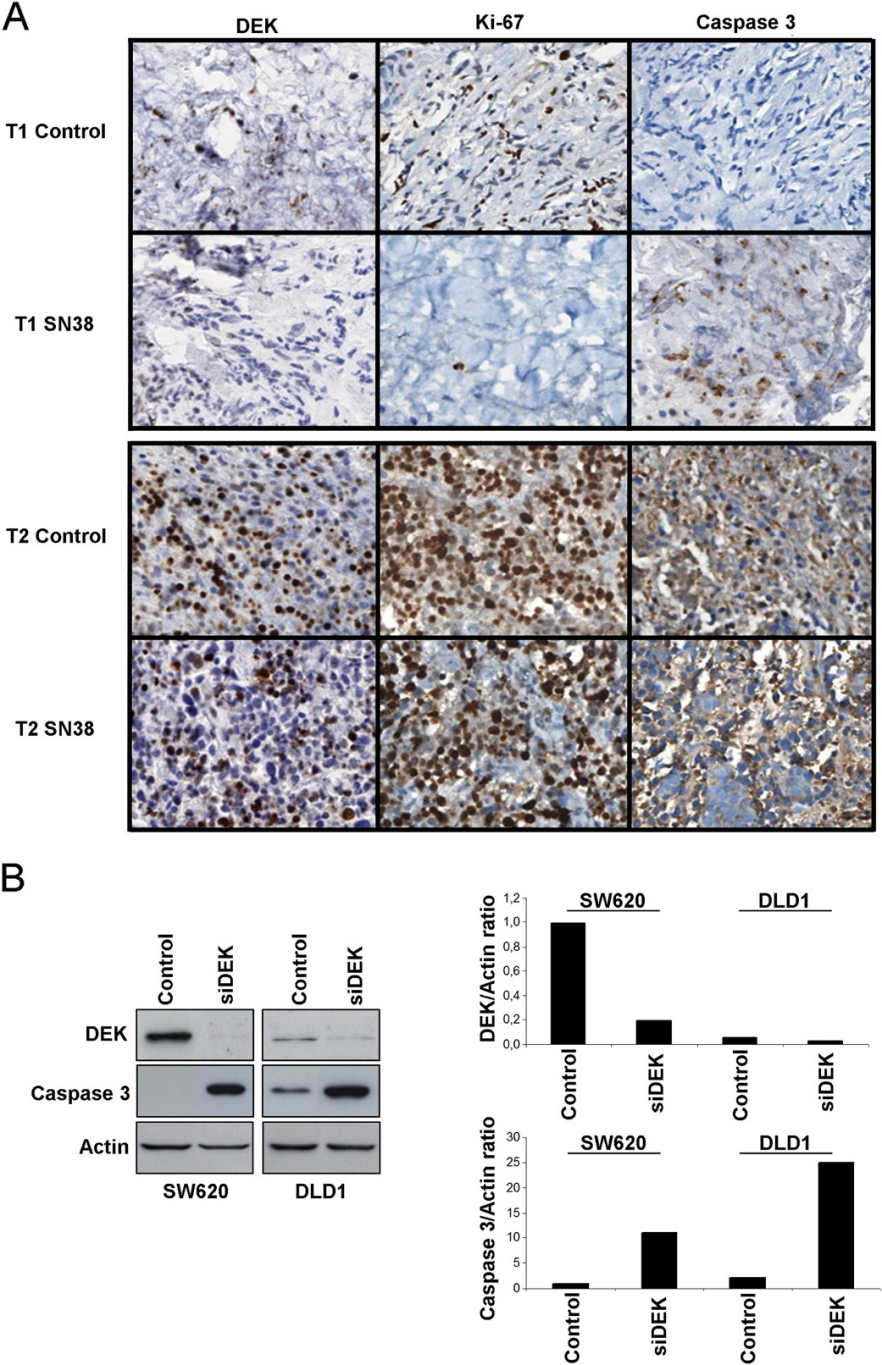
**Low DEK level sensitizes to irinotecan and induces cleaved Caspase 3 expression. A)** *Ex vivo* assay results for the tumor samples with the lowest (T1) and the highest (T2) DEK expression levels. SN38 treatment did not affect DEK expression, but a substantial reduction in Ki-67 and an increase in cleaved-Caspase 3 expression were observed in T1. No changes on Ki-67 or cleaved-Caspase 3 appeared in T2. **B)** Representative Western Blot of DEK, cleaved Caspase 3 and Actin expression after 72 hours of DEK downregulation in SW620 and DLD1 cells with siDEKsec57 (left panel). Densitometric data of Western Blot expressed as ratio of DEK/Actin and cleaved Caspase 3/Actin expression (right panel).

This relation between DEK expression and irinotecan therapy was confirmed in three tumor samples cultured *ex-vivo* with SN38 for 24 hours. Following SN38 treatment, tissues were stained for DEK, Ki-67, and cleaved-Caspase-3. DEK expression was similar between untreated and treated samples indicating that SN38 did not affect its expression. In one of the three patients a substantial reduction in Ki-67 and an increase in cleaved-Caspase-3 expression were observed. This sample corresponds with the one showing the lowest DEK expression levels. The other two tumor samples showed higher DEK expression levels and we did not find differences in any of three analyzed markers (Figure 4A). These results could suggest that DEK level is related to irinotecan response.To assess more deeply the role of DEK level in the induction of the apoptosis, DLD1 and SW620 cells were transfected with siDEK (siDEKsec57) and cleaved Caspase 3 was detected after 72 hours by Western Blot. After DEK downregulation both cell lines increased considerably cleaved Caspase 3 level. Interestingly, cell line with the lowest DEK level, DLD1, showed the highest induction after silencing (Figure 4B). This result suggests the involvement of DEK in apoptosis.

### DEK is a potential predictive marker of survival in *KRAS*^wt^mCRC patients

**Figure 5:**
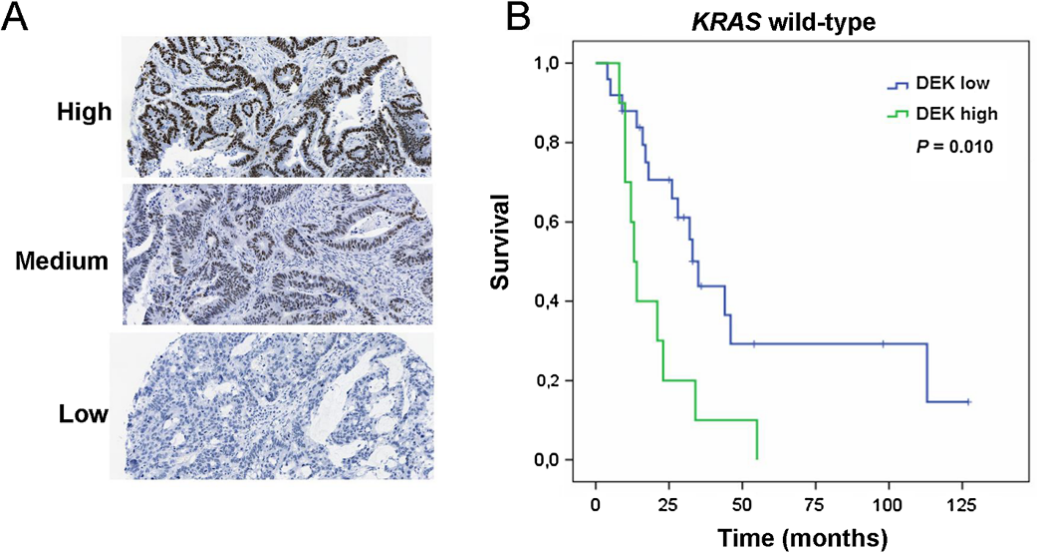
**DEK expression is related to irinotecan response and correlates with poor outcome in** ***KRAS*** ^**wt**^ **patients. A)** Representative IHC images for low, medium or high staining intensities of DEK expression levels. **B)** Kaplan-Meier plot shows a significant association between high DEK level and lower progression-free survival after irinotecan-based treatment in *KRAS* ^wt^ patients (*P* = 0.010).

Based on the previous results suggesting an association between DEK expression and irinotecan sensitivity, we hypothesized that DEK expression levels could be related to the response to irinotecan-based chemotherapy in mCRC. For this purpose, 67 samples from mCRC patients receiving irinotecan-based chemotherapy were selected. Representative images of different DEK expression levels are shown in Figure 5A.

Association between DEK expression levels and progression-free survival after first-line irinotecan-based treatment was analyzed using the Kaplan-Meier method. Survival analysis demonstrated a trend to shorter progression-free survival for patients with higher DEK levels (data not shown). When patients were stratified by *KRAS* mutation status, no correlation between DEK expression and progression-free survival of *KRAS*^mut^ patients was observed (data not shown). However, a significant association with the outcome of patients *KRAS*^wt^ was found (*P* = 0.03, data not shown). The third tertile was established as the best cut-off point, leaving low- and high-risk patient groups (*P* = 0.01, Figure 5B).

Cox regression analysis showed that *KRAS*^wt^ patients with high DEK expression showed increased risk of progression [HR 2.82 (95% CI 1.24-6.45), *P* = 0.01], that remained significant after multivariate analysis [HR 2.4 (95% CI 1.04-5.58), *P* = 0.04] (Table 2).

**Table 2:** **Univariate and multivariate Cox analysis results**

	UNIVARIATE PFS				MULTIVARIATE PFS			
		95% CI				95% CI		
	HR	Lower	Upper	P	HR	Lower	Upper	P
**AGE**	0,966	0,936	0,996	*0,025*	0,971	0,941	1,002	0,068
**CEA**	1,002	0,998	1,005	0,333				
**METASTASIS**				0,398				
Liver	1,000							
Liver & other	1,786	0,571	5,585					
Other	1,872	0,701	4,995					
**BRAF**				0,828				
wild-type	1,000							
mutated	1,119	0,410	3,055					
**BIOLOGIC TREATMENT**				0,763				
None	1,000							
Yes	0,880	0,383	2,022					
**TOPO I**				0,974				
Low	1,000							
High	1,017	0,364	2,845					
**DEK**				*0,014*				*0,040*
Low	1,000				1,000			
High	2,825	1,238	6,449	2,408	1,039	5,579		

Other refers to lung, lymph node and/or peritoneal metastasis.

These results suggest that DEK expression could be a potential predictive marker of sentitivity in KRAS^wt^ mCRC patients receiving irinotecan-based therapy as single first-line treatment.

## Discussion

DEK is a non-histone nuclear protein that performs a transcriptional activity involved in carcinogenesis at multiples levels. In addition, DEK is able to bind cruciform structures and superhelical DNA over linear DNA and introduces positive supercoils [34–36]. Moreover, other studies have shown how nuclear DEK is also able to perform a transcriptional repression of NF-κB pathway through transcriptional repression of cIAP2 and IL-8 in response to TNFα treatment [37]. However, the effect of DEK is not only focused on inflammation but also on neoplasms development and aggressive phenotype maintenance. DEK overexpression has been observed in different tumors [14–21, 23–25, 38].

Our results show DEK downregulation in primary and metastatic CRC human cell lines reduces the migration ability and cell viability, both involved in maintain aggressive phenotype, according to previous studies [27–29, 38].

Irinotecan is a common drug used in clinical practice to treat CRC patients. It is activated by glucuronidation to SN38 and it prevents DNA from unwinding by inhibition of topoisomerase I. After a combination of SN38 treatment and silenced DEK, we observed a significant increase in annexin-V positive cells compared to those treated only with SN38 or DEK knock-down. It is important to note that this effect was not observed after 5FU or LOHP treatments alone or in combination with DEK knock-down. This suggests that low DEK expression sensitizes to SN38.

Regarding to apoptosis process, we observed how DEK downregulation activated Caspase 3. This result correlated with *ex-vivo* assay where an association between low DEK expression and irinotecan sensitivity by induction of cleaved Caspase 3 was found. Lin et al. have been recently reported that silencing of DEK resulted in a decrease in cell proliferation and apoptosis induction revealed by an increase in cleaved Caspase 3 and 9 [38]. These results agreed our data and highlight the involvement of DEK in the proliferation of CRC and its potential role as therapeutic target alone or in combination with irinotecan.

Topoisomerase I expression was determined in most of the samples since it has correlated with irinotecan response in several studies [11, 12] but no association was found between topoisomerase I levels and progression-free survival in this set of patients. Our immunohistochemistry results showed that those mCRC patients with higher levels of DEK expression presented a tendency of shorter progression-free survival after first-line treatment with irinotecan-based chemotherapy. When we stratified patients according to *KRAS* status, we found that *KRAS*^wt^ patients with DEK higher expression had poor outcome independently of topoisomerase I levels. Therefore, these data suggest that DEK is a potential marker of poor prognosis of mCRC patients with *KRAS*^wt^ status.

The reports that involve DEK as a nuclear protein related to cell metabolism by changing supercoiled DNA and maintaining heterochromatin structure along DNA transcription [36] could explain tumor aggressive behaviour and chemoresistance properties. Moreover, we suggest DEK overexpression allows this aggressive phenotype by stabilizing DNA in CRC cells which agrees with higher DEK levels on analyzed metastatic cells lines. We propose that tumor cells induce their apoptosis cascade when two events occur. On one hand, DNA transcription is altered when DEK protein in downregulated and on the other hand, DNA replication is stopped due to topoisomerase I inhibition by irinotecan treatment. For all this, we propose that chemoresistance could be explained by the DNA stabilization properties of DEK.

## Conclusions

The overall data presented here clearly point to a new role of DEK oncogene as a clear factor for the maintenance of the aggressive phenotype in metastatic colorectal cancer and as a potential marker of irinotecan-based therapy response for *KRAS*^wt^ patients.

## Abbreviations

CRC: Colorectal cancer
5FU: 5-fluorouracil
EGFR: Epidermal growth factor receptor
FFPE: Formalin-fixed paraffin-embedded
FOLFIRI: Folinic acid- 5FU-irinotecan
IHC: Immunohistochemistry
MCRC: Metastatic colorectal cancer
PARP-1: Poly-ADP-ribose polymerase 1
siRNA: Small interference RNA sequences
Topo I: Topoisomerase I.
